# Early decrements in bone density after completion of neoadjuvant chemotherapy in pediatric bone sarcoma patients

**DOI:** 10.1186/1471-2474-11-287

**Published:** 2010-12-29

**Authors:** Carsten Müller, Corinna C Winter, Dieter Rosenbaum, Joachim Boos, Georg Gosheger, Jendrik Hardes, Volker Vieth

**Affiliations:** 1 Motion Analysis Lab, Department of General Orthopedics and Tumororthopedics, University Hospital Münster, Domagkstr. 3, 48149 Münster, Germany; 2 Department of Pediatric Oncology and Hematology, University Hospital Münster Albert-Schweitzer.Str. 33, 48149 Münster, Germany; 3 Department of General Orthopedics and Tumororthopedics, University Hospital Münster, Albert-Schweitzer.Str. 33, 48149 Münster, Germany; 4 Department of Clinical Radiology, University Hospital Münster Albert-Schweitzer.Str. 33, 48149 Münster, Germany

## Abstract

**Background:**

Bone mineral density (BMD) accrual during childhood and adolescence is important for attaining peak bone mass. BMD decrements have been reported in survivors of childhood bone sarcomas. However, little is known about the onset and development of bone loss during cancer treatment. The objective of this cross-sectional study was to evaluate BMD in newly diagnosed Ewing's and osteosarcoma patients by means of dual-energy x-ray absorptiometry (DXA) after completion of neoadjuvant chemotherapy.

**Methods:**

DXA measurements of the lumbar spine (L2-4), both femora and calcanei were performed perioperatively in 46 children and adolescents (mean age: 14.3 years, range: 8.6-21.5 years). Mean *Z*-scores, areal BMD (g/cm^2^), calculated volumetric BMD (g/cm^3^) and bone mineral content (BMC, g) were determined.

**Results:**

Lumbar spine mean Z-score was -0.14 (95% CI: -0.46 to 0.18), areal BMD was 1.016 g/cm^2 ^(95% CI: 0.950 to 1.082) and volumetric BMD was 0.330 g/cm^3 ^(95% CI: 0.314 to 0.347) which is comparable to healthy peers. For patients with a lower extremity tumor (n = 36), the difference between the affected and non-affected femoral neck was 12.1% (95% CI: -16.3 to -7.9) in areal BMD. The reduction of BMD was more pronounced in the calcaneus with a difference between the affected and contralateral side of 21.7% (95% CI: -29.3 to -14.0) for areal BMD. Furthermore, significant correlations for femoral and calcaneal DXA measurements were found with Spearman-rho coefficients ranging from ρ = 0.55 to ρ = 0.80.

**Conclusions:**

The tumor disease located in the lower extremity in combination with offloading recommendations induced diminished BMD values, indicating local osteopenia conditions. However, the results revealed no significant decrements of lumbar spine BMD in pediatric sarcoma patients after completion of neoadjuvant chemotherapy. Nevertheless, it has to be taken into account that bone tumor patients may experience BMD decrements or secondary osteoporosis in later life. Furthermore, the peripheral assessment of BMD in the calcaneus via DXA is a feasible approach to quantify bone loss in the lower extremity in bone sarcoma patients and may serve as an alternative procedure, when the established assessment of femoral BMD is not practicable due to endoprosthetic replacements.

## Background

Survival rates in pediatric sarcoma patients have continuously improved within the last decades due to modern multi-agent chemotherapeutic protocols and improvements in diagnostic methods [1,2]. Therefore, the sequelae attributed to the disease or its treatment like a diminished bone mineral density (BMD) are gaining more interest [3] and have been reported previously in pediatric sarcoma patients [4-7]. In this age group a development towards peak bone mass should be observed [8]. Factors negatively influencing BMD in sarcoma patients are polychemotherapy [9], a deficient nutritional status [10], reduced physical activity levels[11,12], and a delayed onset of puberty [13,14] and may correspond with an increased risk of pathologic fractures [15,16]. However, there is still little evidence from current research on the onset and development of bone loss during cancer treatment or on patients being at high risk for diminished BMD during treatment and thus pathologic fractures due to the location of bone sarcomas.

Therefore, the first hypothesis is that children and adolescents undergoing treatment for bone sarcomas reveal BMD decrements already after completion of neoadjuvant chemotherapy. This effect may be ascribed to the disease process and polychemotherapeutic medications.

Secondly, limited mobility for patients with a tumor located in the vicinity of weight-bearing joints in the lower extremity, e.g. pelvis or knee region, may induce more pronounced BMD deficits compared with patients affected in the upper extremity. While neoadjuvant chemotherapy is supposed to have a systemic impact on BMD status, the disease process as well as immobilization in patients with a primary tumor disease in the lower extremity may result in rarefaction and local pathologic bone loss.

Furthermore, patients with a tumor located in the femur or tibia are strongly advised to offload the affected extremity after biopsy due to an increased fracture risk. This is not the case in patients with sarcomas located in the pelvis or the fibula. Hence, we expected significant differences in BMD between the affected and non-affected, contralateral femoral neck and calcaneus with pronounced bone loss in patients with femoral or tibial sarcomas, although the extent of BMD loss in the affected extremity is unknown.

Lastly, the feasibility of peripheral calcaneal BMD assessment via DXA will be evaluated, since the assessment of femoral BMD is often impossible due to endoprosthetic replacements of the proximal femur. Therefore, the objectives of the present study are to evaluate the BMD status in pediatric bone sarcoma patients on completion of neoadjuvant chemotherapy to extract patients being at high risk for low bone density at this early stage of treatment and to evaluate the feasibility of calcaneal DXA measurements.

## Methods

### Patients

Pediatric Ewing's and osteosarcoma patients who met the following criteria were eligible for this cross-sectional study (Table 1): 8-21 years of age, Caucasian origin, and completion of neoadjuvant chemotherapy. Exclusion criteria were: start of adjuvant chemotherapeutic treatment, previous irradiation, and chronic diseases. The study protocol was approved by the local Ethics Committee (2006-216-f-s) and the Federal Office for Radiation Protection (Z5-22462/2-2006-081). All parents and patients, if they attained full age, gave written informed consent.

**Table 1:** Anthropometric and demographic data of the patient group

Participants	Mean (95% CI)
Female/Male	19 (41.3%)/27 (58.7%)
Age (years) at diagnosis	13.9 (13.0 to 14.9)
Age (years) at measurement	14.3 (13.3 to 15.2)
Caucasian	46 (100%)
Height (m)/Z-score	1.64 (1.59 to 1.69)/0.07 (-0.04 to 0.19)
Weight (kg)/Z-score	52.2 (47.5 to 56.9)/-0.31 (-0.51 to -0.10)
BMI (kg/m^2^)/Z-score	18.9 (17.9 to 20.0)/-1.03 (-1.55 to -0.51) *

**Low BMD **(Z-score ≤ -1SD)	**(2 females, 11 males)**
Age (years) at measurement	14.8 (13.1 to 16.4)
BMI (kg/m^2^)/Z-score	18.7 (16.5 to 20.9)/-1.67 (-3.04 to -0.30) *†*
Weight (kg)/Z-score	52.2 (43.7 to 60.7)/-0.68 (-1.24 to -0.12) *†*
**Normal BMD **(Z-score > -1SD)	**(17 females, 16 males)**
Age (years) at measurement	14.1 (12.9 to 15.3)
BMI (kg/m^2^)/Z-score	19.0 (17.7 to 20.4)/-0.44 (-0.89 to 0.0) *†*
Weight (kg)/Z-score	52.2 (46.2 to 58.1)/-0.20 (-0.43 to 0.02) *†*

**Localisation**	**(Female/Male)**
Upper extremity	4 (8.7%)/6 (13.0%) ∑ 10 (21.7%)
Lower extremity	15 (32.6%)/21 (45.7%) ∑ 36 (78.3%)
Femur/Tibia	12 (26.1%)/17 (37.0) ∑ 29 (63.0%)
Pelvis/Fibula	3 (6.5%)/4 (8.7%) ∑ 7 (15.2%)
**Total**	**46 (100%)**

CI = *confidence interval*, BMI = *body mass index*, ∑ = sum, * significant difference between patient group and healthy control group, † significant difference between patients with low BMD (Z-score lumbar spine ≤ -1) and patients with normal BMD status (Z-score lumbar spine > -1)

A reference database [17] was used for the determination of Z-scores for height and weight in patients below the age of 18 years. Z-scores for patients above 18 years of age were calculated by means of an internet database, published by the German Federal Statistical Office [18]. Patients' anthropometrics were comparable to healthy controls with respect to body height that did not differ significantly. The weight of the patient group was slightly lower (not significant), and the body mass index (BMI) was significantly decreased (p = 0.001, Table 1).

### Assessment of Bone Mineral Density (BMD)

Dual-energy X-ray absorptiometry (DXA) scans were performed with a Lunar Prodigy system (enCORE 2006, Software Version 10.51.006, GE Healthcare, UK). Data were automatically compared with the integrated NHANES III Reference Database for the lumbar spine to obtain Z-scores, used in the pediatric population. Z-scores below -2 SD indicate low BMD for a comparable chronologic age [19]. DXA derived BMD values are expressed as areal density (areal BMD in g/cm^2^). These values are dependent on bone size thus introducing potential errors when determining the BMD status of growing children. Therefore, the mathematical model of Kroger et al. [20] was used to calculate volumetric density (volumetric BMD in g/cm^3^). In this model, the vertebral body is considered as a cylindrical shape so that the volume of the cylinder can be calculated using the following formula:

Volumetric BMD=Areal BMD ×[4/(π x width)]

The same applies to the volumetric BMD of the femoral neck by means of the calculation:

Volumetric BMD=Areal BMD×(4/π)*(height/width)

Since Z-scores were only available for the lumbar spine in pediatric patients, reference data published by van der Sluis et al. [21], including areal and volumetric BMD values, were additionally used to evaluate possible decrements in bone density in the patient group.

**Figure 1:**
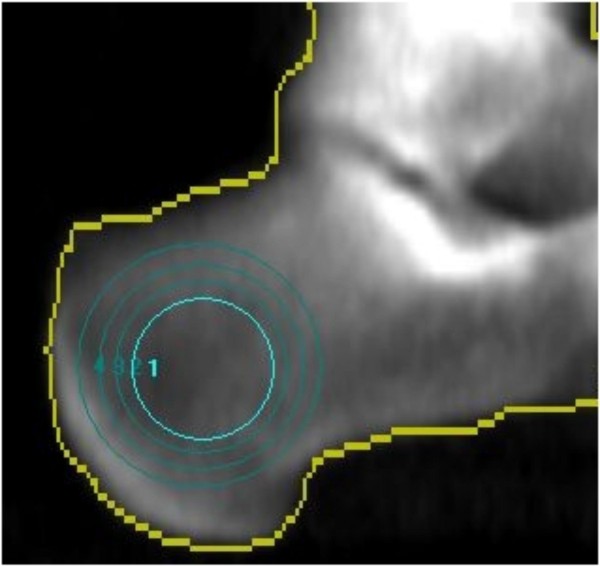
**Implementation of peripheral calcaneal BMD assessment via DXA**.

Calcaneal DXA measurements were facilitated by a modification of the distal radius analysis with repositioning the range of interest (ROI) within the medial calcaneus (Figure 1).

BMD measurements were performed perioperatively on completion of neoadjuvant chemotherapy and before continuing adjuvant chemotherapy in the lumbar spine (L2-4), both femoral necks and both calcanei if feasible. Quality assurance was performed by calibrating the DXA system with a spine phantom supplied by the manufacturer. Coefficients of variation (CV) for repeated measurements were between 0.6% and 0.25%.

### Statistical Analyses

The Mann-Whitney U-test was used for the comparison of BMD of the patient group and reference data from a healthy control group [21]. Mann-Whitney U-test was applied for the comparison of BMD in Ewing's and osteosarcoma patients as well as in male and female patients. Furthermore, the Kruskal-Wallis H-test was applied for the comparison of different primary tumor locations (upper extremity, femur/tibia, pelvis/fibula) and the Mann-Whitney U-Test and Bonferroni correction for pairwise comparisons of the three locations (p value = 0.0167). Wilcoxon signed rank tests were used for the comparison of areal BMD, volumetric BMD and BMC of the affected and non-affected femoral neck and areal BMD and BMC of the affected and contralateral calcaneus for those patients undergoing treatment for a lower extremity sarcoma. Only complete data sets (available for left and right sides) were used for statistical analyses. Furthermore, Spearman correlation coefficients were calculated for the relationship between calcaneal and femoral neck BMD and BMC.

## Results

DXA measurements of the lumbar spine revealed slightly diminished BMD values for the patient group after completion of neoadjuvant chemotherapy with a mean Z-Score of -0.14 (95% CI: -0.46 to 0.18). Z-scores ranged from -2.1 to 2.2. Thirteen patients (28.3%) presented lumbar spine Z-scores below -1 and one patient (2.2%) had low BMD for a comparable chronologic age with a Z-score below -2 (Table 1). Patients with lumbar spine Z-scores below -1 had equal BMI values compared to patients with BMD values greater than -1, but Z-scores for weight and BMI were significantly lower.

Mean values for areal BMD were 1.016 g/cm^2 ^(95% CI: 0.950 to 1.082) and 1.013 g/cm^2 ^(95% CI: 0.960 to 1.066) for the patient group and the reference data group [21], respectively. Differences between both groups were not statistically significant. Calculated volumetric BMD values again revealed no significant differences in the lumbar spine BMD for the comparison of the patient group (vBMD = 0.330 g/cm^3^, 95% CI: 0.314 to 0.347) with reference data (vBMD = 0.328 g/cm^3^, 95% CI: 0.318 to 0.339) [21].

The comparison of lumbar spine BMD in upper extremity (mean Z-score -0.44, 95% CI: -0.94 to 0.06) and lower extremity sarcoma patients (mean Z-score -0.06, 95% CI: -0.45 to 0.34) revealed no significant difference. However, Ewing's sarcoma patients, whose neoadjuvant treatment lasts 18 weeks and includes six cycles of chemotherapy, revealed a significant lower lumbar spine Z-score (-0.61, 95% CI: -1.15 to -0.07) compared with osteosarcoma patients (0.14, 95% CI: -0.25 to 0.52, p = 0.016), whose neoadjuvant treatment lasts ten weeks with two cycles of chemotherapy.

Mean lumbar spine Z-scores were lower in males with -0.37 (95% CI: -0.78 to 0.03) than in female patients with 0.19 (95% CI: -0.34 to 0.72) but did not differ significantly. Only one female patient, undergoing treatment for a Ewing's sarcoma located in the acetabulum, had a Z-score lower than -2 and hence low bone density already after completion of neoadjuvant chemotherapy. In addition, we found the lowest lumbar spine BMD values in patients undergoing treatment for pelvic (Z-score -1.08, 95% CI: -1.98 to -0.17) or upper extremity sarcomas (Z-score -0.44, 95% CI: -0.94 to 0.06, Table 2).

**Table 2:** Effect of tumor location on BMD expressed as Z-scores and percent compared to healthy age and gender matched controls

	Mean (95% CI)	Mean (95% CI)	*p-value*
	Lumbar spine Z-score	Age comparison [%]	
Upper Extremity (n = 10)	-0.44 (-0.94 to 0.06)	94.5 (88.4 to 100.6)	0.393 *
Lower Extremity (n = 36)	-0.06 (-0.45 to 0.34)	99.2 (94.4 to 103.9)	

Upper Extremity (n = 10)	-0.44 (-0.94 to 0.06)	94.5 (88.4 to 100.6)	0.284 †
Femur/Tibia (n = 29)	0.03 (-0.39 to 0.45)	100.2 (95.3 to 105.2)	
Pelvis/Fibula (n = 7)	-0.40 (-1.72 to 0.92)	94.7 (78.2 to 111.2)	

CI = *confidence interval*

* Mann-Whitney U-test, † Kruskal Wallis H-test

**Figure 2:**
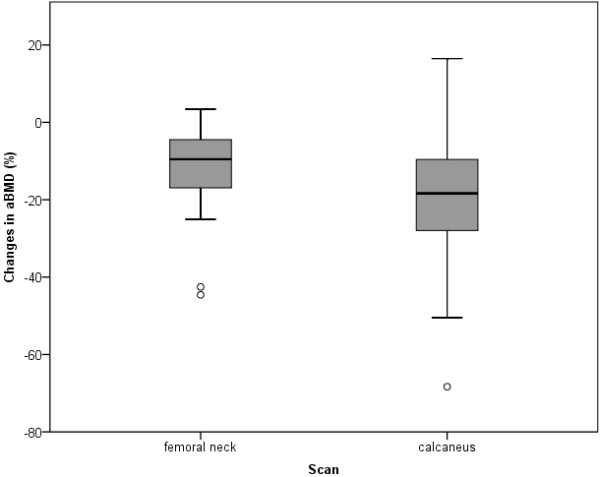
**aBMD differences between affected and contralateral sides of the femoral neck and calcaneus.** Boxplots represent median, 25^th ^and 75^th ^percentile, 1.5 interquartile range (IQR) and outliers

The third hypothesis was that BMD decrements may occur locally as a consequence of offloading recommendations in patients with primary tumor locations in the lower extremity. Therefore, the BMD status in the affected and non-affected, contralateral sides of the femoral neck and calcaneus were evaluated. Wilcoxon signed-rank tests revealed significant decrements (p < 0.001) in the affected compared with the non-affected, contralateral side of the femoral neck and the calcaneus (Table 3). Decreased BMD values were more pronounced in the calcaneus (p = 0.014) with -21.7% (95% CI: -29.3 to -14.0) for areal BMD and -21.7% (95% CI: -29.4 to -14.1) for BMC than in the femoral neck with -12.1 (95% CI: -16.3 to -7.9) for areal BMD and -11.1 (95% CI: -16.9 to -5.3) for volumetric BMD (Figure 2). Furthermore, mean percent changes in BMD between affected and contralateral femoral neck and calcaneus were greatest for patients with sarcomas located in the weight-bearing bones, i.e. femur and tibia (Table 4). They accounted for 13% between both femoral necks and 23% between both calcanei. Femoral and calcaneal BMD in the affected extremity declined to a lesser extent in patients with primary tumor locations in the pelvis or the fibula (not significant).

**Table 3:** Mean BMD and BMC values of the affected and contralateral femoral neck and calcaneus for lower extremity sarcoma patients

Scan region	Affected Mean (95% CI)	Contralateral Mean (95% CI)	p-value*
**FN (n = 29)**			
aBMD (g/cm^2^)	0.877 (0.794 to 0.959)	0.993 (0.922 to 1.064)	p < 0.001
vBMD (g/cm^3^)	0.358 (0.328 to 0.388)	0.402 (0.384 to 0.420)	p < 0.001
BMC (g)	4.18 (3.66 to 4.69)	4.75 (4.22 to 5.28)	p < 0.001

**Calc (n = 25)**			
aBMD (g/cm^2^)	0.457 (0.391 to 0.523)	0.583 (0.524 to 0.641)	p < 0.001
BMC (g)	1.47 (1.26 to 1.68)	1.87 (1.69 to 2.06)	p < 0.001

BMD = *bone mineral density*, aBMD = *areal bone mineral density (g/cm^2^)*, vBMD = *volumetric bone mineral density (g/cm^3^)*, BMC = *bone mineral *content, CI = *confidence interval*, FN =*femoral neck*, Calc = *calcaneus*

* Mann-Whitney U-test

**Table 4:** Mean percent difference in BMD between affected and contralateral side in sarcoma patients

			Mean (95% CI)	Mean (95% CI)	Mean (95% CI)
	Location	n=	aBMD (g/cm^2^)	vBMD (g/cm^3^)	BMC (g)
**FN**	UpEx	10	-1.3 (-5.8 to 3.3)	-3.2 (-10.2 to 3.9)	3.3 (-5.6 to 12.1)
	Femur/Tibia	22	-13.7 (-18.8 to -8.5)	-12.7 (-20.1 to -5.3)	-13.6 (-18.1 to -9.1)
	Pelvis/Fibula	7	-7.1 (-14.6 to 0.4)	-6.2 (-13.8 to 1.5)	-7.8 (-15.5 to 0.0)

**Calc**	UpEx	8	-4.4 (-9.3 to 0.4)		-4.3 (-9.0 to 0.5)
	Femur/Tibia	19	-22.5 (-30.1 to -15.0)		-22.6 (-30.1 to -15.1)
	Pelvis/Fibula	6	-18.8 (-47.9 to 10.3)		-18.9 (-47.8 to 10.0)

* Lower extremity sarcoma patients subgrouped into sarcomas within the vicinity of primary weight-bearing joints (femur/tibia) and pelvis/fibula.

CI = *confidence interval*, aBMD = *areal bone mineral density (g/cm^2^)*, vBMD = *volumetric bone mineral density (g/cm^3^)*, BMC = *bone mineral content (g)*, UpEx = *Upper Extremity*, FN =*femoral neck*, Calc = *calcaneus*

Post-surgery tumor-endoprosthetic replacements or difficulties in patient positioning for bone density analysis via DXA may complicate the assessment of femoral BMD or even prevent DXA application. Therefore, the feasibility of peripheral BMD assessment of the calcaneus was assessed. Data obtained from femoral and calcaneal sites were correlated to evaluate whether this approach may serve as an alternative BMD assessment with peripheral DXA in a population of pediatric sarcoma patients. Both, the affected and contralateral sides of the femoral neck and calcaneus were significantly correlated with Spearman-rho correlation coefficients ranging from ρ = 0.55 and ρ = 0.80 (Table 5).

**Table 5:** Spearman correlation coefficients for BMD of the femoral neck and calcaneus in patients with a primary tumor located in the lower extremity (n = 24)

Bone Density	ρ =	*p-value*
aBMD affected	0.654	0.001
aBMD contralateral	0.798	< 0.001
BMC affected	0.549	0.005
BMC contralateral	0.741	< 0.001
vBMD_femur _- aBMD_calcaneus _affected	0.649	0.001
vBMD_femur _- aBMD_calcaneus _contralateral	0.694	< 0.001

BMC = *bone mineral content*, aBMD = *areal bone mineral density*, vBMD = *volumetric bone mineral density*

## Discussion

To our knowledge this is the first study determining the BMD status of children and adolescents undergoing treatment for bone sarcomas with respect to local osteopenia conditions. All enrolled patients received neoadjuvant chemotherapy according to the protocols of Euramos-1 (osteosarcoma) and Euro-E.W.I.N.G.99 (Ewing's sarcoma) thus representing a comparatively homogenous patient group within each protocol. After completion of neoadjuvant chemotherapy no significant decrease in lumbar spine BMD was observed. Obtained data rather indicate that bone loss occurs primarily in terms of focal osteopenia within the affected extremity. This is confirmed by the significantly decreased BMD of -12.1% of the femoral neck when comparing the affected and contralateral side. On the other hand, differences in BMD of the calcaneus between affected and contralateral sides were significantly higher and accounted for -21.7%.

Unexpected, only one female patient with a low lumbar spine BMD status after completion of neoadjuvant chemotherapy for a bone sarcoma located in the acetabulum was found. However, patients with a tumor located in the pelvis revealed the lowest lumbar spine BMD with an average Z-score of -1.08. Z-scores for the whole patient group indicate that there is only a slight, but non-significant reduction in lumbar spine BMD in this early stage of treatment. Most probable, bone formation is already decreased after completion of neoadjuvant chemotherapy, but BMD decrements using DXA may not yet be detectable.

Patients with primary tumor locations in the lower extremity were expected to present lower lumbar spine Z-scores due to adhering to offloading recommendations or impaired gait as a consequence to tumor related pain and subsequently reduced physical activity levels. In fact, patients with upper extremity tumors revealed lower lumbar spine Z-scores of -0.44 compared to Z-scores of patients treated for lower extremity sarcomas with -0.06, even though the difference between both groups was not significant.

Previous studies on BMD in sarcoma patients focused on survivors and found reduced BMD values [4,5,7,22]. Kaste et al. [7] examined 99 survivors of rhabdomyosarcoma, osteosarcoma and Ewing's sarcoma at least one year after completion of polychemotherapy and found a decreased BMD status with a median Z-score of -0.75. Low BMD was correlated with younger age at diagnosis and treatment with cyclophosphamide in rhabdomyosarcoma patients. Neither medications with methotrexate (MTX), ifosfamide nor endocrinopathies were correlated with diminished BMD values. Findings of a higher risk for low bone density in patients with primary upper extremity sarcomas compared with lower extremity tumor patients were confirmed in the present study.

Holzer et al. [4] found reduced BMD values in two thirds of 48 adult survivors of osteosarcoma: 44% revealed osteopenic and 21% osteoporotic values. Ruza et al. [5] reported an even higher incidence of osteopenia in 60% and osteoporosis in 27% of 63 osteosarcoma and Ewing's sarcoma patients six years after diagnosis. This indicates that BMD loss is likely to occur in pediatric bone sarcoma survivors; it may develop in the long run and persist for several years. Longitudinal studies are necessary to define the occurrence of diminishing bone density. Therefore, the assessment of BMD should already be incorporated during the acute treatment phase and should be repeated every six months, since sarcoma patients are at increased risk for developing low BMD during and after treatment. The reasons are likely to be multifactorial and include extensive adjuvant chemotherapy, radiation, premature gonadal hormone failure, and reduced physical activity levels. However, serial densitometry screens by means of DXA in pediatric patients being at risk for low BMD are difficult to interpret, since they depend on gender, body size, and pubertal stage [13,23,24].

High doses of methotrexate are part of neoadjuvant chemotherapy in osteosarcomas. This agent is known to negatively affect bone metabolism by means of increasing bone resorption and to inhibit bone formation by affecting the differentiation of early osteoblastic cells [25]. Therefore, osteosarcoma patients were assumed to reveal lower BMD values than patients undergoing treatment for Ewing's sarcoma. This could not be confirmed in the present cross-sectional study. Ewing's sarcoma patients had significantly lower lumbar spine Z-scores. Most likely the difference between both diagnosis groups is due to a significantly shorter duration of neoadjuvant treatment in osteosarcoma patients with two cycles of chemotherapeutic agents (methotrexate, adriamycin and cisplatin) in ten weeks in comparison to 18 weeks of neoadjuvant treatment and six cycles VIDE (vincristine, ifosfamide, doxorubicin and etoposid/cisplatin) in Ewing's sarcoma patients. While ten weeks of treatment do not seem to be linked with a significant loss of lumbar spine BMD in osteosarcoma patients, neoadjuvant treatment with nearly twice the time and more than twice the dose intensity in Ewing's sarcoma patients induced significant lumbar spine BMD. Longitudinal studies with particular focus on osteosarcoma patients would help to clarify this hypothesis. However, our findings are in accordance with previous studies by Arikoski et al. [26,27] who demonstrated normal BMD values for the lumbar spine and femoral neck at diagnosis in a heterogeneous group of pediatric patients with different cancer entities, even though bone formation was already decreased. The diminishing effect on BMD due to impaired bone turnover was not shown until six months of polychemotherapy [28].

The results of the present study demonstrate that bone loss exists in pediatric sarcoma patients after neoadjuvant chemotherapy in terms of focal osteopenia. Regional low BMD status can be explained as an effect of offloading recommendations for patients with a lower extremity sarcoma. Since the calcaneus is mainly composed of trabecular bone that is known to react faster to metabolic changes than cortical bone, the reduced load on the affected leg and in particular on the calcaneus is supposed to result in a rapid reduction of BMD [29]. Thus, the present results confirm a recent study quantifying postoperative bone loss in children [30]. Regional lower BMD is attributable to prolonged immobilization and limited weight-bearing. The awareness of a tumor in the lower extremity leads to a cautious movement behaviour with a further load reduction. This finding of local BMD loss in pediatric patients with solid cancer is in accordance with previous findings [6].

This study extends available information on BMD decrements in pediatric sarcoma patients by determining the differences between affected and non-affected lower extremity bone density. Sarcoma patients experience a mean regional bone loss of up to 13% in the femoral neck and 23% in the calcaneus already after preoperative chemotherapy. The highest difference for femoral areal BMD of 45% was found in a male patient treated for Ewing's sarcoma located in the distal femur. After local therapy, usually consisting of tumor surgery, lower extremity sarcoma patients have to continue offloading the affected extremity. Patients undergoing tumor surgery for a pelvic sarcoma are confined to bed for at least six weeks. Cementless reconstructions of the femur afford offloading of the affected leg with a subsequent gradually increase of the load of ten kg per week. After recovery from surgery, sarcoma patients show reduced activity levels due to restrictions to the ward and gait impairments [31]. This implies that bone strength is lost as a consequence of immobilization [32]. Therefore, the extent of BMD loss in the affected leg has to be determined in future longitudinal studies. New strategies like promotion of physical activity while undergoing cancer treatment as well as an immediate reintegration after cessation of tumor therapy will have to be established to prevent significant bone loss due to cancer treatment. It should be evaluated whether exercise interventions are feasible in sarcoma patients and which loads are essential for the affected extremity.

Furthermore, the present results reveal that peripheral DXA measurements performed at the calcaneus can serve as an alternative for the generally established assessment of BMD in the femoral neck region, especially in cases where femoral DXA measurements are not feasible due to common problems in pediatric bone sarcoma patients like positioning difficulties or endoprosthetic replacements.

Finally, several limitations of this study have to be acknowledged. The first refers primarily to the patient group. Although only bone sarcoma patients were enrolled in this study, they represent a fairly heterogeneous sample due to different age groups (8-21 years) and treatment protocols (Euramos-1 and Euro-E.W.I.N.G.99). While the Euramos-1 protocol for osteosarcoma patients consists of ten weeks of neoadjuvant treatment in which methotrexate, adriamycin and cisplatin are administered, patients within the Euro-E.W.I.N.G. 99 protocol receive six cycles VIDE (vincristine, ifosfamide, doxorubicin and etoposid/cisplatin) of neoadjuvant chemotherapy prior to local therapy within 18 weeks on average.

Furthermore, treatment-related delay of pubertal maturation may have a substantial impact on BMD in bone sarcoma patients [14]. Therefore, the lack of a Tanner stage assessment allows no unequivocal statement about whether a delayed maturation leads to BMD decrements in bone sarcoma patients.

The implementation of peripheral DXA assessment of calcaneal BMD was not performed with a pDXA instrument as described in literature [33], but a whole body axial scanner. Since our intention was not the development of reference values but the determination of changes in BMD between the affected and contralateral extremity, using an axial DXA scanner was appropriate. Finally, due to a lack of pediatric anthropometric data from the German Federal Statistical Office we had to refer to a second database for patients below the age of 18 years [17],

## Conclusions

In summary, childhood and adolescence represent a critical time with respect to attaining peak bone mass. Treatment for bone sarcomas may interfere with the accumulation of an appropriate peak bone mass and hence may lead to an increased fracture risk in adulthood, constituting a significant reduction in quality of life. Exercise intervention programs are essential and should begin immediately after diagnosis. Screening patients at risk for low BMD should already start at the beginning of cancer treatment and be repeated in regular intervals of six to twelve months. Peripheral BMD evaluation of the calcaneus is a relevant and appropriate procedure, in particular when established femoral neck DXA measurements are not feasible due to common endoprosthetic replacements in sarcoma patients.

## Abbreviations

aBMD: areal bone mineral density (g/cm^2^); BMC: bone mineral content; BMD: bone mineral density; BMI: body mass index; Calc: calcaneus; CI: confidence interval; CV: coefficient of variation; DXA: dual-energy x-ray absorptiometry; FN: femoral neck; IQR: interquartile range; L2-4: lumbar vertebrae 2-4; ROI: range of interest; UpEx: upper extremity; vBMD: volumetric bone mineral density (g/cm^3^)

## Competing interests

The authors declare that there are no competing interests.

## Authors' contributions

CM was involved in data collection, analyses and manuscript preparation; CCW was involved in data collection and analyses; JH was involved in manuscript revision; DR, JB, GG and VV initiated the study and were involved in manuscript revision. All authors read and approved the final manuscript.

## Pre-publication history

The pre-publication history for this paper can be accessed here:

http://www.biomedcentral.com/1471-2474/11/287/prepub
